# Which environmental factors are associated with lived health when controlling for biological health? - a multilevel analysis

**DOI:** 10.1186/s12889-015-1834-y

**Published:** 2015-05-27

**Authors:** Cristina Bostan, Cornelia Oberhauser, Gerold Stucki, Jerome Bickenbach, Alarcos Cieza

**Affiliations:** Department of Health Sciences and Health Policy, University of Lucerne, Lucerne, Switzerland; Swiss Paraplegic Research, Nottwil, Switzerland; Department of Medical Informatics, Biometry and Epidemiology–IBE, Pettenkofer School of Public Health (PSPHLMU), Research Unit for Biopsychosocial Health, Ludwig Maximilian University (LMU), Munich, Germany; Faculty of Social and Human Sciences, School of Psychology, University of Southampton, Southampton, UK

**Keywords:** Lived health, Biological health, Environmental factors, Multilevel Item Response Theory, Spain

## Abstract

**Background:**

Lived health and biological health are two different perspectives of health introduced by the International Classification of Functioning, Disability and Health (ICF). Since in the concept of lived health the impact of the environment on biological health is inherently included, it seems intuitive that when identifying the environmental determinants of health, lived health is the appropriate outcome. The Multilevel Item Response Theory (MLIRT) model has proven to be a successful method when dealing with the relation between a latent variable and observed variables. The objective of this study was to identify environmental factors associated with lived health when controlling for biological health by using the MLIRT framework.

**Methods:**

We performed a psychometric study using cross-sectional data from the Spanish Survey on Disability, Independence and Dependency Situation. Data were collected from 17,303 adults living in 15,263 dwellings. The MLIRT model was used for each of the two steps of the analysis to: (1) calculate people’s biological health abilities and (2) estimate the association between lived health and environmental factors when controlling for biological health. The hierarchical structure of individuals in dwellings was considered in both models.

**Results:**

Social support, being able to maintain one’s job, the extent to which one’s health needs are addressed and being discriminated against due to one’s health problems were the environmental factors identified as associated with lived health. Biological health also had a strong positive association with lived health.

**Conclusions:**

This study identified environmental factors associated with people’s lived health differences within and between dwellings according to the MLIRT-model approach. This study paves the way for the future implementation of the MLIRT model when analysing ICF-based data.

## Background

National health surveys have become a widely used tool to collect household, family and person-level data on health status and risk factors since the middle of the last century. The continuous information on health and many of the factors that affect health enable many hypotheses to be tested. In the United States, the National Health and Nutrition Examination Survey has monitored health for over 50 years [1]. In England, the Health Survey has been running annually since 1991 [2]. While the household component of these surveys collects limited demographic information on all of the individuals living in a particular household, the family and person component collects data on topics including health status and limitations, health care services and health behaviors.

The information collected in these surveys is based on very diverse instruments and methodologies, which makes cross-country comparisons very challenging. A recent review of all instruments used in national surveys from 18 Western European countries, as well as from Canada, Australia and the United States, showed that standardised health assessment is needed for comparability purposes [3].

One approach to achieve standardisation and comparability of health data increasingly discussed in the scientific literature is to use the International Classification of Functioning, Disability and Health (ICF) as a basis for developing survey tools [4, 5]. The ICF is the World Health Organisation’s standard for the description of functioning and health. Its framework has been used to collect standard health information in national health surveys in Spain [6], Italy [7], Ireland [8], Chile [9] and Mexico [10].

By using of the ICF for data-collection purposes at the population level, the assessment of two perspectives of health is being introduced, namely what we refer to as ‘biological health’ and ‘lived health’. In the ICF, the ‘under the skin’ perspective of health–or biological health–is captured by the ICF concept of capacity. The lived health perspective–the outcome of the interaction between one’s level of capacity and the positive or negative impact of one’s environment–is captured by the concept of performance. The concept of lived health requires a full consideration of the environment’s effect on people’s lives [11]. Environmental factors include the physical environment (the physical world and it’s features, and the human-made physical world), the social environment (different relationships and roles with other people, people’s attitudes), social systems and services, and policy, rules and laws [4]. A scoping review of peer-reviewed studies with the focus on the effect of the environment on lived health of children and youth with disabilities showed that the common facilitators of higher levels of lived health are social support of family and friends and positive attitudes within family and community. In contrast, the most common barriers are negatives attitudes within family and community, followed by the physical accessibility of the environment and the lack of support from service providers [12]. Raggi et al. also showed that in case of neurological conditions, non-employed people showed more than twice the odds of being severely disabled [13].

Since in the concept of lived health the impact of the environment on health is inherently included, it seems intuitive that when identifying the environmental determinants of health using survey data, lived health is the appropriate outcome. Having information about both biological health and lived health makes it possible in regression models to control for biological health when lived health is the sought outcome. In this way, a more appropriate estimation of the real impact of the environment can be calculated because it allows us to control for differences in the intrinsic bodily capacity of individuals in the target population. When included in population-health surveys, the concepts of biological health and lived health can be captured in terms of single domains of function, such as mobility, self-care or communication, which can then be integrated into a single score. This has been previously done in other investigations [14, 15]. The literature shows that it would lead to biased estimates of environmental determinants to include those scores in linear regression models since one would be considering the dependent variable in fixed terms and disregarding the nested structure of the sample. Therefore, it would be of advantage to make use of an approach that allows biological and lived health to be viewed as random terms. This is something that can be achieved with the Multilevel Item Response Theory (MLIRT) model. This model also has the advantage of taking into consideration the hierarchical structure of the data when estimating the biological health and lived health single scores. This model has been successfully used in educational research for studying the observed factors associated with the educational effectiveness in schools, but not yet in health research [16]. Overall experience of health cannot be directly assessed. Therefore, obtaining an estimate (score) of overall health based on several responses to questions on bodily impairments, activity limitations and participation restrictions is challenging [17]. In addition, validly comparing health state for different health conditions and across different regions is challenging [18]. This paper introduces a new approach for analysing data based on the conceptual framework of the ICF. Countries which have already implemented the ICF in population-based health surveys or which are planning to implement it would benefit from this approach.

The objective of this investigation is, therefore, to identify environmental factors associated with lived health when controlling for biological health using the MLIRT framework. The intention is also to facilitate current and future data-analysis efforts using the ICF in population-health surveys.

## Methods

### Ethical aspects

This analysis was conducted on a de-identified, public-use data set. According to Spanish legislation, no approval of an ethics committee was necessary.

### Study design and participants

We used the community-dwelling population data from the Spanish 2008 Survey on Disabilities, Independence and Dependency Situations (EDAD 2008). This survey was carried out throughout the entire country and was aimed to estimate the number of people with disabilities residing in Spain in main family dwellings [6]. The EDAD 2008 design has been described previously [19]. In brief, first a Spanish representative sample of family dwellings was drawn. A family dwelling represents a room or a set of rooms and their outbuildings which are located in a building destined to be inhabited by one or several households. A household is a person or a group of persons who share food and others goods paid for within the same budget.

All households within the dwelling were independently included in the study. A total of 91,290 households were then selected after the main informant was identified and asked whether s/he agreed for all household members to be screened for the study’s disability criterion, namely to have ‘important limitations’ in at least one of eight domains: *seeing, hearing, communication, learning and application of knowledge and development of tasks, mobility, self-care, home life, interactions and interpersonal relationships*. ‘Important limitation’ was defined as a limitation ‘in carrying out everyday activities that has lasted or is expected to last for longer than one year and whose origin is an impairment’. A total of 21,583 persons over 6 years of age fulfilled the study’s disability criterion and were included in the survey. Data were collected based on individual face-to-face interviews filling in printed questionnaires.

### Variables

Forty-two questions were used to assess the level of difficulty in carrying out activities without any technical aid or personal assistance. We considered these questions to be biological health questions. Thirty-one questions assessing the level of difficulty in most of the same activities, but taking into account any kind of technical aid or personal assistance, were also asked. We consider these questions to assess lived health. Each lived health question was only answered by people who for each health domain reported the use of technical aids, personal assistance or both. For all the others, lived health in each domain was equated with biological health. The ordinal scale used to assess the limitation level consisted of the following response options: 1 = Without difficulty or with little difficulty; 2 = With moderate difficulty; 3 = With severe difficulty and 4 = Cannot carry out the activity.

Additional questions were asked about: persons’ characteristics; medical conditions; health, social and economic benefits; changes in economic status; social networks and contacts; and personal care received. Health, social and economic benefits questions included in this study refer to 1) the use by the person with disabilities of socio-health services in the last 14 weeks or 12 months and 2) economic benefits in the last 12 months as a result of some disability: periodic benefits (e.g., pensions due to disability, life and disability insurance, benefits through the company in which people work), non-periodic benefits (e.g., public aid for education, rehabilitation), compensations due to physical injury and tax benefits in personal income taxes or other taxes. Information on dwelling accessibility was also collected.

### Data analysis

Questions referring to vision and hearing were not considered because no differentiation was made between with and without assistive devices or personal assistance. Furthermore, only adults who had difficulty in at least one of the biological health questions were included in the analyses. As a result, 17,303 adults living in 15,263 different dwellings were kept in the analyses.

A descriptive analysis of the study population was carried out, taking sampling weights into account. The response options ‘with moderate difficulty’ and ‘with severe difficulty’ in both biological health and lived health questions showed a low frequency. Thus, we collapsed them into one single option called ‘with moderate/severe difficulty’.

To identify the environmental factors associated with lived health when controlling for biological health using the MLIRT framework, we used (1) the MLIRT model to calculate a biological health score for each person and (2) the MLIRT model to study the association between environmental factors and the lived health score when controlling for the biological health score calculated in step 1. The MLIRT model was implemented in the R software by Fox [16]. This model integrates the Graded Response Model (GRM) that measures the individual scores with a structural, multilevel model that explains the differences between the lived health score of people living within the same dwelling (person level) and people living in different dwellings (dwelling level) since we considered the hierarchical structure of individuals in dwellings in the structural model. Two separate item parameters are estimated in a GRM model: item thresholds (or response option difficulties) and item discrimination. Item thresholds indicate the location on the latent trait where the item best discriminates between individuals. For a person with a fixed value on the latent trait, the item thresholds provide the information which response option is the most likely for that person on an item. If the person ability is below/above the threshold, the person is most likely to respond in the lower/higher response option. The item discrimination parameter indicates how well an item discriminates between individuals, especially between individuals with abilities close to its thresholds. The higher the discrimination parameter, the better the item discriminates between individuals.

We used this model due to its ability to: (1) avoid biased parameter estimates in the structural multilevel model by integrating the error associated with the ability parameters resulting from the GRM (ability considered as random) in the model, (2) allow the integration of exploratory variables at different levels of hierarchy and at the person and dwellings levels, (3) deal with incomplete data, i.e., variation across individuals in terms of completed questions is allowed, and the number of individuals across dwellings may vary; missing values were treated as missing completely at random and (4) provide more accurate estimates in ability parameter and item parameter estimates when including additional covariates in the estimation procedure.

In the first MLIRT model, we estimated the individual’s biological health scores based on biological health question responses. Based on the concept of biological health, we didn’t include any person and dwelling exploratory variable into the structural model. However, the random effects for the dwellings were included.

In the second MLIRT model, we estimated the lived health scores taking into account the environmental factors that determine lived health. In the structural part of the MLIRT model, age, gender, biological health, discrimination due to one’s health condition, the capability to maintain one’s job and the extent to which a person’s health needs are addressed were taken as person-level observed variables. The availability of environment adaptation of the dwelling was taken as a dwelling-level observed variable. Again, the dwelling information was included as a random variable.

To study whether the IRT model could be used for our data, we evaluated the IRT model assumptions–unidimensionality, local independency and monotonicity–separately for biological health and lived health questions. Unidimensionality was examined with bifactor analysis using analytic bifactor rotations [20, 21]. The assumption of unidimensionality is supported when all questions load highly on the general factor. Local independency was tested by examining the residual correlations among questions in a single-factor model confirmatory factor analysis [22, 23]. We estimated the MLIRT model with and without the flagged local dependent questions (residual correlations higher than 0.25) to see if estimates (people’s posterior means) were robust to question dependencies. Monotonicity was studied by examining graphs of the question mean scores conditional ‘rest-scores’ (i.e., total raw scale score minus the question score). This IRT assumption was satisfied if the probability of selecting a response option indicating difficulty in carrying out an activity is higher if the level of difficulty across the other activities is higher. Questions that failed one of these three assumptions were not considered in the final model [24].

In the MLIRT models, the Markov Chain Monte Carlo (MCMC) scheme developed by Fox and Glas was used to estimate all model parameters simultaneously [25]. In each MCMC iteration, the means and variance of the vector of sampled abilities were fixed at zero and one, respectively. The convergence and the burn-in period for the convergence were estimated using boa software [26]. For each of the estimates, the posterior means over the posterior density distribution of the estimates, the posterior standard deviations and the 95% highest posterior density intervals (HPD) are presented. The HPD is an interval within which most of the posterior distributions of the estimates lie. We also provided the model’s deviance information criterion (DIC) [27] for each model. The DIC is an indicator of the model fit and of the model complexity. The smaller the DIC, the better the model will predict a replicate data set which has the same structure as the one currently used.

All analyses were performed with R [28].

## Results

Table 1 shows the characteristics of the study population. Sixty percent of the respondents were more than 65 years old.

**Table 1:** Characteristics of the community-dwelling population

	Community-dwelling population (*N* = 17303)	
	N	%
Gender		
Female	10881	62.4
Male	6422	37.6
Age, years		
Younger than or equal to 65 years	6748	40.4
Older than 65 years	10555	59.6
Education		
No school	2008	11.4
Primary school incomplete	6141	35.0
Primary school complete	5242	29.3
Secondary school first step	1566	9.3
Secondary school finished	851	5.4
Professional school medium	455	2.9
Professional school superior	276	1.7
University	740	5.0
Number of health conditions^a^		
No health condition	2572	14.9
One health condition	4703	27.2
Two health conditions	3957	23.0
Three or more health conditions	6071	35.0

All data are population weighted

^a^The health conditions were: Spinal-cord injury, Parkinson’s disease, Lateral sclerosis, Multiple sclerosis, Agenesis/Amputation, Laryngectomy, Arthritis, Rheumatoid arthritis or Ankylosing spondylitis, Muscular dystrophy, Spina bifida/hydrocephalus, Myocardial infarction or Ischaemic cardiomyopathy, Cerebrovascular accidents, Down’s syndrome, Autism and other disorders associated with autism, Cerebral palsy, Acquired brain damage, Senile Dementia of the Alzheimer Type, Other types of dementia, Schizophrenia, Depression, Bipolar disorder, Retinitis pigmentosa, Myopia magna, Senile macular degeneration, Diabetic retinopathy, Glaucoma, Cataract, HIV/AIDS, Rare illnesses, Cancer

### Biological health scores

The results from bifactor analysis of biological health questions showed that questions from the *mobility* domain loaded higher on their respective group factor than on the general factor. We decided to proceed with unidimensional biological health since this domain contributes to biological health and no substantial difference in people’s posterior means was observed when estimating the model without the mobility questions.

The left part of Table 2 shows the local dependent questions, as well as the questions considered in the final models. The monotonicity assumption was not satisfied by two questions, namely: ‘With what level of difficulty would you say you are able to carry out activities related to menstrual care?’ and ‘With what level of difficulty would you say you are able to drive vehicles?’.

**Table 2:** Biological health and lived health questions included in the MLIRT models and their parameter estimates for the final MLIRT models

	Biological health				Lived health			
Questions	Discr	HDP	Thr1	Thr2	Discr	HDP	Thr1	Thr2
Communication								
With what level of difficulty would you say you are able to:…								
Speak intelligibly or utter coherent phrases?^(l,1)*^	1.593	[1.543, 1.652]	1.942	3.308	0.865	[0.831, 0.902]	1.459	2.396
Understand what other persons say to you?^(l,1)^	1.739	[1.663, 1.804]	2.139	3.769				
Understand and express yourself in writing?	1.937	[1.851, 2.016]	2.179	3.013				
Understand and express yourself via gestures, symbols, illustrations or sounds?	2.449	[2.359, 2.524]	3.112	4.442				
Hold a dialogue and exchange ideas with one or more persons?	2.167	[2.074, 2.250]	2.471	3.965				
Use the telephone or other devices or communication techniques?^(l,1)^	1.455	[1.391, 1.501]	1.636	2.165				
Learning and application of knowledge and development of tasks								
With what level of difficulty are you able to:…								
Hold a gaze or pay attention when listening?	1.904	[1.837, 1.965]	2.527	4.109				
Learn to perform simple tasks?	2.090	[2.008,2.191]	2.412	3.239				
Perform simple tasks?^(l,2)*^	2.435	[2.304,2.532]	2.945	3.880	1.491	[1.430, 1.557]	2.306	3.019
Perform complex tasks?^(l,2)*^	1.982	[1.895, 2.049]	2.084	2.830	1.106	[1.072, 1.139]	1.584	2.209
Mobility								
At what level of difficulty are you able to:…								
Change posture?^(b,1)^					1.448	[1.406, 1.488]	0.106	3.240
Keep your body in the same position?^(b,1)^					1.306	[1.266, 1.342]	−0.108	2.802
Walk and move around the home?^(b,1)*^	1.205	[1.164, 1.242]	0.087	1.716	1.727	[1.666, 1.773]	0.304	3.438
Walk or move outside the home?^(b,2)*^	1.056	[1.021, 1.084]	−0.391	1.194	1.456	[1.421, 1.487]	−0.142	2.498
Get around via public transport?^(b,2)^					1.377	[1.341, 1.411]	−0.062	2.021
Drive vehicles?								
Lift or carry objects?^(b,3)^					1.239	[1.206, 1.268]	−0.181	1.742
Handle and move objects?^(b,3)^					1.511	[1.473, 1.547]	0.035	2.319
Lift or carry small objects?^(b,3)*^	1.158	[1.122, 1.196]	0.213	1.721	1.624	[1.577, 1.667]	0.246	2.487
Self-Care								
With what level of difficulty would you say are you able to:…								
Wash or dry different body parts?	2.690	[2.608, 2.781]	0.191	2.317	2.552	[2.471, 2.624]	0.908	3.840
Perform basic grooming?	2.474	[2.401, 2.544]	0.231	2.054	2.239	[2.167, 2.330]	0.918	3.040
Carry out activities related to urination?	2.423	[2.355, 2.492]	0.554	2.829	1.987	[1.933, 2.059]	0.957	3.358
Carry out activities related to defecation?	2.608	[2.499, 2.766]	1.059	3.118	2.401	[2.331, 2.462]	1.449	4.062
Carry out activities related to menstrual care?								
Dress or undress?	2.916	[2.790, 3.034]	0.417	2.914	2.881	[2.810, 2.940]	1.294	4.495
Eat and drink?	2.299	[2.176, 2.393]	1.212	3.653	2.118	[2.033, 2.203]	1.739	4.596
Follow medical prescriptions?	3.201	[3.134, 3.285]	0.818	2.899	1.725	[1.660, 1.772]	1.437	2.905
Avoid dangerous situations?	3.218	[3.044, 3.385]	1.182	3.178	2.368	[2.294, 2.425]	1.464	3.170
Home Life								
At what level of difficulty would you say are you able to:…								
Do shopping?^(b,4)^					1.505	[1.469, 1.535]	0.082	1.154
Prepare meals?	1.837	[1.794, 1.880]	0.329	1.161	1.893	[1.842, 1.937]	0.555	1.483
Carry out housework?^(b,4)*^	1.375	[1.337, 1.410]	−0.173	0.812	1.661	[1.616, 1.700]	0.140	1.297
Interaction and interpersonal relations								
With what level of difficulty would you say are you able to:…								
Show others affection, respect or transmit feelings?	1.741	[1.657, 1.812]	2.421	3.726				
Relate to strangers?	1.928	[1.848, 2.017]	2.294	3.060				
Initiate and maintain relationships with subordinates, peers or superiors?	1.982	[1.919, 2.042]	2.471	3.134				
Initiate and maintain relationships with friends, neighbours, acquaintances or colleagues?	1.827	[1.761, 1.884]	2.325	3.233				
Initiate and maintain family relations?^(b,5)^								
Initiate and maintain intimate or sexual relations?^(b,5)*^	1.644	[1.564, 1.730]	1.993	2.367				

The subsets of local dependent questions are marked with (letter, number), with the letter indicating whether the local dependent questions are biological health questions (‘b’) or lived health questions (‘l’) and the number indicating the subset of local dependent questions. The questions of each subset of local dependent questions considered in the final model are marked with an asterisk *

Discr = item discrimination; Thr 1–2 = item threshold parameters; HPD = 95% highest posterior density interval

The left part of Table 2 shows the item parameter estimates. The most discriminating question was ‘avoid dangerous situations’ (with a discrimination of 3.218). This means that this question differentiates well between people with different levels in biological health. The least discriminating question was ‘walk or move outside the home’ (with a discrimination of 1.056). The question for which only individuals in the worst biological health are expected to have high difficulties is ‘understand and express yourself via gestures, symbols, illustrations or sounds’ (with a threshold of 4.442 on the logit scale).

**Fig. 1:**
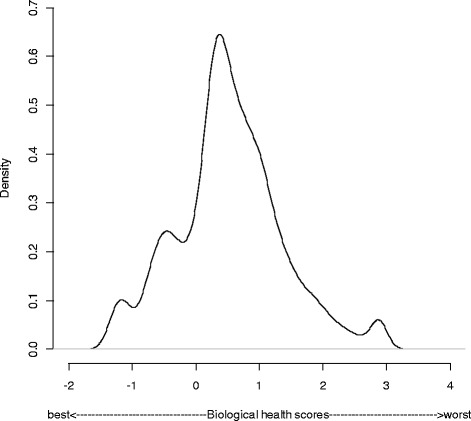
Density curve showing the distribution of the standardized biological health score expressed in logits and taking sampling weights into account

Figure 1 shows the distribution of standardized biological health scores taking sampling weights into account. Values for biological health range from −1.682 to 3.022, with higher values indicating worse biological health.

### Environmental factors associated with lived health when controlling for biological health

When testing the unidimensionality IRT assumption for lived health, the bifactor analysis showed higher loadings of the communication lived health questions on their group factor than on the general factor. Since these questions contribute to the measure of lived health and the posterior means didn’t differ when estimating the model with and without communication lived health questions, we decided to keep these questions in the MLIRT model.

The right part of Table 2 shows the local dependent questions, as well as the questions considered in the final model. Only questions from the communication domain were not considered after checking for robustness to question dependencies when estimating the MLIRT models with and without the flagged local dependent questions. Monotonicity was graphically supported by all the items with the exception of the questions measuring the level of difficulty in carrying out activities related to menstrual care and driving vehicles. While the most discriminating question was ‘dress or undress’ (with a discrimination of 2.881), the least discriminating question was ‘speak intelligibly or utter coherent phrases’ (with a discrimination of 0.865). The question for which only individuals in the worst lived health are expected to have high difficulties is ‘eat and drink’ (with a threshold of 4.596 on the logit scale). Values for lived health range from −1.906 to 3.265, with higher values indicating worse lived health.

Table 3 presents the structural model parameter estimates of the MLIRT model with person-level and dwelling-level explanatory variables. Biological health had a strong positive association with lived health. Being male or aged younger than 65 years was significantly associated with less difficulty in carrying out the activities of daily living. The experience of being discriminated against because of one’s disability, any economic benefit received due to disability and the daily contact with family and friends enable people to carry out their daily activities and, therefore, to have higher levels of lived health. Moreover, a person’s job has is an important factor since people who change their work activity or profession or are not working tend to have lower levels of lived health.

**Table 3:** Parameter estimates of the MLIRT model using environmental variables as possible explanatory variables of the lived health score when controlling for biological health

	MLIRT model		
	Mean	SD	HPD
Fixed effects	−0.072	0.016	[−0.098, −0.042]
Intercept			
*Person-level variables*			
Biological health	0.902	0.005	[0.891, 0.912]
Gender (Ref: Female)	−0.172	0.009	[−0.188, −0.154]
Age (Ref: Younger)	0.101	0.011	[0.080, 0.123]
Health and social services			
Needed and not received in the last 14 days (Ref: Not needed)	0.068	0.021	[0.028, 0.105]
Needed and received in the last 14 days (Ref: Not needed)	0.057	0.009	[0.038, 0.072]
Needed and not received in the last 12 months (Ref: Not needed)	0.091	0.031	[0.038, 0.150]
Needed and received in the last 12 months (Ref: Not needed)	0.078	0.008	[0.060, 0.093]
Economic benefit			
Received in the last 12 months (Ref: Not received)	−0.075	0.016	[−0.108,-0.047]
Change in work activity and profession			
Change in work activity in the same profession (Ref: No change)	0.028	0.048	[−0.074, 0.112]
Change in occupation or profession (Ref: No change)	0.269	0.035	[0.199, 0.340]
Change in relation to the current work activity and occupation (Ref: No change)	0.148	0.013	[0.123, 0.172]
Maintaining one’s job			
Being out of work in the last 3 months (Ref: Currently working)	−0.102	0.059	[−0.234, −0.003]
Being out of work between 3 months and one year (Ref: Currently working)	0.067	0.039	[−0.012, 0.135]
Being out of work between one and 5 years (Ref: Currently working)	0.097	0.022	[0.052, 0.139]
Being out of work more than 5 years (Ref: Currently working)	0.037	0.017	[0.004, 0.070]
Disability discrimination			
Feeling discriminated due to disability (Ref: Not feeling discriminated)	−0.103	0.014	[−0.131, −0.076]
Social relationship			
Having daily contact with family (Ref: No contact)	−0.047	0.010	[−0.069, −0.028]
Having daily contact with a friend (Ref: No contact)	−0.022	0.009	[−0.039, −0.007]
Personal care			
Receive personal assistance or care due to disability (Ref: Not received)	0.008	0.013	[−0.015, 0.031]
*Dwelling-level variables*			
No availability of environment adaptation of dwelling (Ref: Availability)	0.002	0.007	[−0.013, 0.015]

SD = Standard deviation; HPD = 95% highest posterior density interval

Only 15% of the variance in people’s lived health was explained by grouping people into dwellings. To judge on model fit, we additionally estimated the empty MLIRT model in which no exploratory variable besides the intercept was considered. The results showed a better fit of the model to the data when exploratory variables are included (DIC = 447424.6 for the empty MLIRT model vs DIC = 426687.1 for the final MLIRT model). The proportion of variance explained by exploratory variables was 77% on the person level and 74% on the dwelling level.

## Discussion

In this investigation, we identified the following environmental factors associated with health operationalized as lived health when controlling for biological health and using the MLIRT model: social support, work-related factors, the extent to which one’s health needs are addressed and discrimination due to one’s health problems.

There are specific open questions that require further discussion, both with the identified environmental factors associated with lived health and the statistical model. Environmental factors identified as associated with lived health cover a broad range of health determinants that have been described in former publications [29]. We showed that economic benefits or compensations as a result of some disability are positively associated with people’s lived health. This result confirms the conclusion of Rodriguez-Laso et al. that economic difficulties are associated with negative daily experiences of lived health [30]. Whether a person receives economic benefits or not, the Spanish health and social-security systems support people with health problems so that they can keep their jobs or enter into a different profession [31, 32]. Our study showed that people who change professions tend to have lower levels of lived health compared with people who do not, even when controlling for biological health. This result emphasises the need for vocational-rehabilitation programs to help people with health problems to maintain their jobs. We also showed that people who are away from their work for longer than one year have poorer lived health, even when controlling for biological health. Thus, it is also important to implement programmes for returning to work as early as possible [33, 34].

This study provided evidence that health is positively associated with variables related to social support. Prior research in Spain has shown that the higher the frequency of contacts with family members or friends, the lower the probability of having health problems [35]. This may be related to attitudes of family or friends in the Spanish society. In Spain, people with health problems have daily contact with others more often than in other European countries [36].

A growing body of research suggests that experiencing discrimination because of health problems can have a negative effect on one’s health [37]. However, feeling discriminated produces a paradox, that might be called ‘discrimination paradox’. We showed that feeling discriminated is associated with higher levels of lived health. This result was also confirmed in a study based on data from the European social survey covering 26 countries. Alvarez-Galvez et al. showed that people from Spain, Norway, Sweden and Denmark who reported experiencing discrimination were also likely to report positive health outcomes. While in Scandinavian countries this association could be explained by the well-developed welfare-state system, additional research is needed to explain the same association in Spain [38].

Regarding the statistical approach used in this study, we employed the MLIRT model for each step of the analysis to calculate a biological health score and identify environmental factors associated with lived health when controlling for biological health. Since we used the R package mlirt, which does not allow for the integration of two IRT models for the estimation of both the biological health score and the lived health score, we had to calculate the biological score separately from the lived health score and calculate a second model for the lived health score in which the biological health score was included as a known predictor (error fixed to zero). This could affect the estimates of the second MLIRT model. However, we looked at the nested structure of the data for estimating the biological health score. We recognise that it would have been better to treat biological health as a random variable and to perform both analytical steps in one, but this possibility is the only one allowed in the software S-plus, which is not publicly available. Besides the MLIRT model’s advantages mentioned in the method section, it is important to emphasize that correlations between the lived health abilities and environmental factors did not affect the accuracy or precision of the threshold and the discrimination parameters of lived health questions [39].

Epidemiologic research provided evidence that a person’s lived health at one point in time is the result of the cumulative effect of exposures during one’s life span [40]. However, it is a challenge to measure how society ‘got into the body’ over time. With the inclusion of the biological health score in the model, the impact of the environment on the body over the life span is implicitly taken into account. Our study also confirmed that biological health is an important predictor of lived health. We carried out the analysis without controlling for biological health, and the results showed worse fitting of the model to the data (DIC = 440725.5).

### Strengths and limitations

The most important strength of this study is its large nationally representative Spanish sample. However, the Spanish Disability Survey has several limitations that need to be mentioned. Firstly, from the national representative sample only people with at least one ‘important’ limitation in functioning were asked about their biological health and lived health. Thus, the sample and, consequently, our results are only representative of people with limitations in functioning and not the general population. Secondly and related to the previous limitation, when transforming categorical data to a metric scale, the ‘important’ difficulty became the lowest response option value. This decision could affect the comparability of our results with other studies that used different thresholds on the health continuum. This also emphasises the need to avoid thresholds when collecting population-based data [41]. Thirdly, the Spanish study was cross-sectional, and it was not possible to discuss its findings in terms of causal relationships. Another limitation is related to a general limitation of the MLIRT model, namely more accurate item estimates are obtained at smaller test lengths and sample sizes. However, the large sample size used in our study minimized the bias obtained in item parameters [39].

## Conclusions

This investigation provides evidence obtained by implementing the MLIRT model that non-biased parameter estimates can be retrieved when studying the environmental factors associated with lived health while controlling for biological health using data from ICF-based population surveys. This study paves the way for the future implementation of the MLIRT model when analysing ICF-based data. Interventions aimed at enhancing health in community-dwelling populations can be designed and their effectiveness investigated more accurately when non-biased statistical results are available.

## Abbreviations

ICF: International Classification of Functioning, Disability and Health
MLIRT: Multilevel Item Response Theory
GRM: Graded Response Model
IRT: Item Response Theory
HPD: 95% highest posterior density interval
DIC: Deviance Information Criterion
EDAD: Survey on disabilities, independence and dependence situations
